# Updated Insights on EGFR Signaling Pathways in Glioma

**DOI:** 10.3390/ijms22020587

**Published:** 2021-01-08

**Authors:** Alexandru Oprita, Stefania-Carina Baloi, Georgiana-Adeline Staicu, Oana Alexandru, Daniela Elise Tache, Suzana Danoiu, Elena Simona Micu, Ani-Simona Sevastre

**Affiliations:** 1 Department of Biochemistry, Faculty of Medicine, University of Medicine and Pharmacy, 200349 Craiova, Romania; opr.alexandru@gmail.com (A.O.); carina_baloi@yahoo.com (S.-C.B.); adstaicu@gmail.com (G.-A.S.); dodolita@yahoo.it (D.E.T.); 2 Department of Neurology, University of Medicine and Pharmacy of Craiova and Clinical, Hospital of Neuropsychiatry, 200349 Craiova, Romania; oanale@hotmail.com; 3 Department of Pathophysiology, Faculty of Medicine, University of Medicine and Pharmacy, 200349 Craiova, Romania; suzdanoiu@gmail.com; 4 Department 4—Medical Specialities, Faculty of Medicine, University of Medicine and Pharmacy, 200349 Craiova, Romania; 5 Department of Pharmaceutical Technology, Faculty of Pharmacy, University of Medicine and Pharmacy, 200349 Craiova, Romania

**Keywords:** glioma, pathways, EGFR, clinical trials

## Abstract

Nowadays, due to recent advances in molecular biology, the pathogenesis of glioblastoma is better understood. For the newly diagnosed, the current standard of care is represented by resection followed by radiotherapy and temozolomide administration, but because median overall survival remains poor, new diagnosis and treatment strategies are needed. Due to the quick progression, even with aggressive multimodal treatment, glioblastoma remains almost incurable. It is known that epidermal growth factor receptor (EGFR) amplification is a characteristic of the classical subtype of glioma. However, targeted therapies against this type of receptor have not yet shown a clear clinical benefit. Many factors contribute to resistance, such as ineffective blood–brain barrier penetration, heterogeneity, mutations, as well as compensatory signaling pathways. A better understanding of the EGFR signaling network, and its interrelations with other pathways, are essential to clarify the mechanisms of resistance and create better therapeutic agents.

## 1. Introduction

With an overall survival of less than 35% in five years [1], malignant primary brain tumors are the most difficult to treat cancers. Of those, the most common type is represented by gliomas. Based on the expression patterns’ differences, glioblastomas are divided into three subtypes as follows: classical, proneural, and mesenchymal [2]. Because glioblastoma multiforme (GBM), a grade IV glioma [3], is one of the most aggressive primary brain tumors, recent studies and reviews have focused on deepening our understanding of the disease [4,5,6,7,8,9].

At present, GBM’s pathogenesis is better understood due to recent advances in molecular biology. For newly diagnosed glioblastoma, the current standard of care is represented by resection, followed by radiotherapy and temozolomide (TMZ) administration [10], but the median overall survival (OS) is not fully improved; therefore, new diagnosis and treatment strategies are needed [11,12].

Glioblastoma is the most common and the most deleterious glioma [13]. The 2011–2015 Statistical Report of the Central Brain Tumor Registry of the United States (CBTRUS) showed that glioblastoma represents 48% of the malignant brain and central nervous system tumors, with an incidence rate in the United States 1.58 times higher in males compared to females [14]. Due to the quick progression, even with aggressive multimodal treatment, glioblastoma remains almost incurable [15,16].

Nowadays, chemotherapy has a significant role in glioblastoma’s treatment strategies, with numerous research studies aimed to develop more efficient chemotherapeutic drugs [17]. Understanding the disease’s pathogenesis has a key role in identifying disease biomarkers and developing new potential chemotherapeutic drugs. We present some of the most promising signaling pathways involved in pathogenesis, with their specific targeting components.

GBM is characterized by nuclear atypia, cellular pleomorphism, mitotic activity, anaplasia, and rapid proliferation alternated with an aggressive invasion of the surrounding brain tissue. In its microenvironment, glioma cells are faced with many challenges such as acidity, hypoxia, and low nutrient availability. To maintain rapid growth, they need to modulate metabolic activity [18,19].

In multicellular organisms, tyrosine phosphorylation is involved in signal transduction, leading to differentiation, proliferation, migration, and survival [20,21].

Receptor tyrosine kinases (RTKs) are activated by binding their extracellular domain to corresponding ligands determining their oligomerization. This process activates the intracellular domain, facilitating the recruitment of proteins that start a signaling cascade, integrating numerous signaling pathways that lead to specific cellular responses [22]. Among all RTKs, epidermal growth factor receptor (EGFR) is the most amplified in GBM [23]. EGFR amplification is observed in the classical subtype of glioma [2]. EGFR gene amplification is detected in 57.4% of primary GBM patients, leading to high levels of EGFR protein, contributing to tumorigenesis and progression [24].

However, targeted therapies against this type of receptor have not yet shown a clear clinical benefit. Many factors contribute to resistance, such as ineffective blood–brain barrier penetration, heterogeneity, mutations, and compensatory signaling pathways. A better understanding of the EGFR signaling network and its interrelations with other pathways are essential to improve drug activity, clarify the mechanisms of resistance, and develop better therapeutic agents.

## 2. Understanding EGFR Features

The transmembrane receptor of tyrosine kinase epidermal growth factor (EGFR), also known as HER (human EGFR related) 1 or ErbB1, along with HER2/neu (ErbB-2), HER3 (ErbB-3), and HER4 (ErbB-4), is a member of the ErbB family and it is located on chromosome band 7p12 [25].

Like all RTKs, EGFR has an extracellular region, a single transmembrane domain, an intracellular juxtamembrane domain, a tyrosine kinase, and a C-terminal region. The ligands of ErbB receptors are divided into two main groups: EGFR activators called EGF agonists, and neuregulins that bind to ErbB3 and ErbB4 [26,27].

The extracellular region of EGFR has two homologous domains (I and III) that bind ligands and two cysteine rich domains (II and IV) [28].

The juxtamembrane region tethers inactive EGFRs to the plasma membrane cytosolic surface, which contributes to EGFR activation [29]. Structural studies highlight the functional importance for certain regions, such as the structure of the first 30 amino acids from the intracellular juxtamembrane region of EGFR and the C-terminal 190 amino acids [27].

There are more than 40 EGFR ligands that control its signaling. They can be divided into high-affinity ligands, such as epithelial growth factor (EGF), heparin-binding EGF-like growth factor (HB-EGF), Transforming growth factor alpha (TGF-α), betacellulin (BTC), and low-affinity ligands, such as epiregulin (EREG), amphiregulin (AREG), and epigen (EPGN).

Expression of EGF-family proteins and activation of EGFR are features of cardiac disease [30,31]. Moreover, molecular alterations of EGFR include overexpression, deletion, or amplification, in different types of cancer. In GBM, EGFR amplification promotes invasion, proliferation, and drug resistance to radio- and chemotherapy [32].

Several trials on EGFR targeted therapy have failed to produce conclusive evidence, maybe because of the EGFR molecular heterogeneity in GBM, of the low specificity of the designed drugs, as well as because of low brain penetration [33]. Despite all this, the detection of EGFR alterations is still used as a prognostic marker for GBM because 24–67% of GBMs are characterized by a mutated gene, 40% by amplification, and 60% by EGFR overexpression [34].

In recent years, studies have proved that EGFR has pro-survival kinase-independent functions in malignant cells. This fact has offered a different perspective of understanding EGFR implications in cancer, with new ideas of EGFR targeted cancer therapy [35,36,37].

## 3. Mechanisms of EGFR Pathway Activation

There are several different mechanisms of EGFR pathway activation, such as increased ligand production or overexpression/defective inactivation/mutation of the receptor. Many studies focused on the EGFR signaling mechanism in recent years, trying to conclude how the extracellular EGFR-ligand binding propagates through the single transmembrane helix (TM) to trigger intracellular kinase activation [38,39,40].

### 3.1. EGFR Activation Mechanisms in Normal Physiologic Status

The expression of EGFR in normal cells is about 4 × 10^4^–10 × 10^4^ receptors/cell [41], whereas, in cancer cells, more than 10^6^ receptors/cell are observed [42]

The EGFR RNA expression is increased by stimulating the EGFR-specific transcription factor (ETF). The receptor expression is regulated by epidermal growth factor (EGF) itself and other proteins such as E1A, Sp1, and AP2 [36].

Like all RTKs, EGFR is activated by ligands featuring receptor-specificity. Briefly, ligand binding leads to a dimeric active conformation of EGFR by homodimerization (complexed with another EGFR) or heterodimerization (complex with another ErbB member). The tyrosine residues from other RTKs are autophosphorylated after ligand stimulation, and phenylalanine substitutions significantly impair the kinase signaling and the downstream signaling. Differently, EGFR Tyr-845 phosphorylation is not a required mechanism for ligand-induced EGFR activation, but it may represent the main mechanism for EGFR transactivation [43,44].

Proteins that express a proto-oncogene tyrosine-protein kinase (Src) homology domain 2 (SH2) region bind to the activated receptor, areactivated, and forward the signal to the downstream effectors, propagating critical cellular signaling pathways [45]. EGFR can simultaneously activate several signal transduction pathways such as phosphatidylinositide 3 kinase (PI3K) and serine–threonine kinase (AKT) and RAS/MAPK pathways [46].

#### 3.1.1. Extracellular Domain Activation

For EGFR, the dimerization is completely receptor-mediated, with no physical interaction between two activating ligands. In normal physiologic status, the receptors are in a dynamic monomer–dimer equilibrium. In the absence of ligands, the extracellular domain presents a tethered configuration (intra-molecular links entirely block the dimerization arm), and the intracellular tyrosine kinase domain (TKD) is inactive. Ligand binding leads to a conformational change that exposes the buried dimerization arm, and the extracellular domain dimerizes, inducing conformational changes of the intracellular domain and enabling kinase activation [45].

A recent study by Chung et al. described physiological EGFR activation as being due to a ligand-mediated extracellular domain dimerization that stabilizes the *N*-terminal transmembrane dimer and disrupts autoinhibition, allowing the C-terminal juxtamembrane (JM-B) segment to stabilize the asymmetric kinase domain (KD) dimer, resulting in activation of EGFR signaling. They also concluded that the stimulus stabilizes the active KD conformation in pathological states and further the asymmetric KD dimerization. The inside–out coupling is weaker than the physiological outside–in coupling, suggesting that the extracellular (EC) dimer is linked through the *N*-terminal TM dimer with the asymmetric oncogenic KD dimer [38].

#### 3.1.2. Intracellular Domains Activation

By ligand-induced dimerization, the cis-autoinhibition is released, and through a unique allosteric mechanism, the kinase activity of EGFR isactivated. It is well known that this mechanism consists of physical interaction between the C-terminal tail of the activator kinase and the other kinase *N*-terminal tail (receiver kinase) of the dimer pair, inducing conformational changes of the *N*-lobe of receiver kinase and trans-phosphorylation C-terminal tail of the activator [47].

#### 3.1.3. Downstream Signaling of EGFR

**Figure 1:**
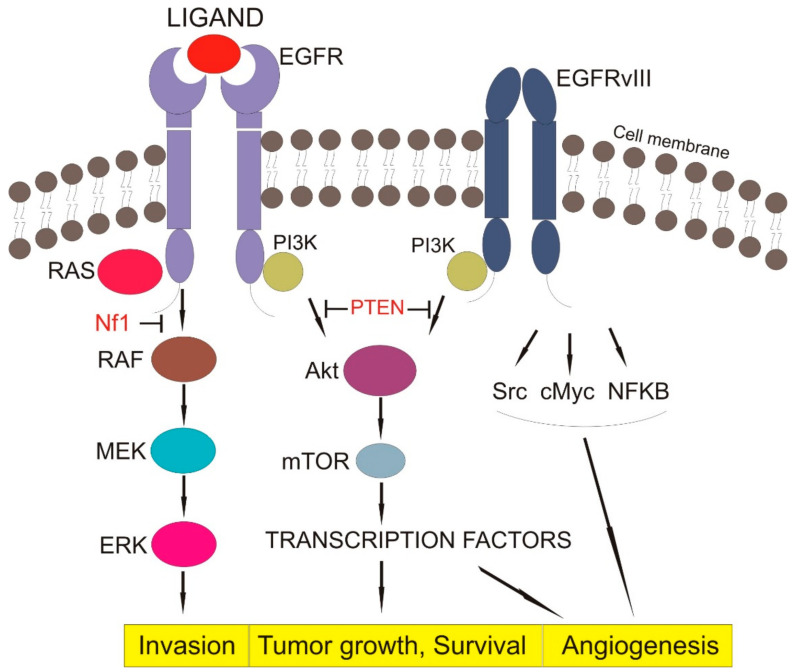
EGFR signaling pathway (EGFR—epithelial growth factor receptor, EGFRvIII—Epidermal growth factor receptor variant III, Pi3K—Phosphoinositide 3-kinase, RAS—family of genes involving cellular signal transduction, PTEN—Phosphatase and tensin homolog, NF1—Neurofibromatosis type 1, RAF—serine/threonine-specific protein kinases, MEK—Mitogen-activated protein kinase, ERK—extracellular signal-regulated kinase, AkT—Protein kinase B, mTOR—mammalian target of rapamycin, Src—Proto-oncogene tyrosine-protein kinase, cMyc—c proto-oncogene, NFKB—nuclear factor kappa-light-chain-enhancer of activated B cells, Block arrow—inhibition activity, Point arrow—pathway flow).

EGFR activation and autophosphorylation result in the recruitment of downstream signaling proteins. Almost all autophosphorylation sites are binding sites for Src Homology 2-(SH2) or Phosphotyrosine binding-(PTB) signaling proteins. The SH2- proteins may be bound directly to the receptor, or indirectly through docking proteins using PTB domains [48]. EGFR can recruit and regulate many signaling pathways such as PI-3 K/AKT, RAS/MAPK, and JAK2/STAT. Therefore, EGFR functions as a hub involved in regulating various cellular processes [21,23], as shown in Figure 1.

The PI-3K/AKT signaling pathway involves PI3K, an enzyme with SH2-signal transducer and its downstream effector AKT, regulating apoptosis and cell survival. Once EGFR is activated and phosphorylated, PI3K is brought to the cell membrane, and it phosphorylates phosphatidylinositol 4,5-bisphosphate (PIP2), forming phosphatidylinositol (3,4,5)-trisphosphate (PIP3). AKT reacts with PIP3, and it is phosphorylated at Threonin308 by phosphoinosite-dependent protein kinase-1 (PDK1) and at Serine 473 by the mammalian target of rapamycin complex 2 (mTORC2), reaching full activity. The phosphatase and tensin homolog (PTEN) negatively regulate the PI3K/AKT pathway by dephosphorylating and delocalizing PIP3 from the cellular membrane, resulting in the relocalization of AKT in the cytoplasm, where it is unable to be reactivated [49,50].

Class IA is one of the three different classes of PI3Ks featuring subunits with regulatory activity such as p85. Active EGFR achieves association with regulatory p85 through dimerization with human HER3, or via the docking protein GRB2-associated binder 1 (GAB1), relieving the inhibitory effect of p85 [51]. GAB1 is a scaffolding protein involved in recruiting additional signaling proteins such as PI3K, SHP2, and p120RasGap. It is involved in many EGFR signaling outputs, and is the predominant mechanism linking EGFR to PI3K/Akt signaling [52,53].

Due to its increasing importance in different human cancers, GAB1 may represent an emerging potential therapeutic target.

The RAS/MAPK signaling pathway involves the growth-factor-receptor bound-2 (GRB2), which forms a complex with Son of Sevenless (SOS), a guanine-nucleotide exchange factor (GEF) and activates the RAS G-protein by exchanging guanosine diphosphate (GDP) with guanosine triphosphate (GTP) [54]. Consequently, RAS and mitogen-activated protein kinases (MAPKs) initiate a downstream signaling cascade to phosphorylate the nuclear protein Jun. Jun creates complexes with different nuclear proteins leading to the key transcription factor activator protein 1 (AP-1), responsible for translation and transcription of proteins involved in the growth and division of cells. Activated RAS is negatively regulated by GTPase activating proteins (GAPs), such as the tumor suppressor neurofibromin 1 (NF1) [55].

Signal transduction and activator of transcription 3 (STAT3) is tyrosine-phosphorylated or activated as pSTAT3 due to EGFR-regulation of interleukin-6 (IL-6) expression. This mechanism leads to a feed-forward in the IL-6/Janus kinase (JAK)/STAT3 loop [21,56,57,58].

### 3.2. Oncogenic Status and EGFR Activation

The EGFR is one of the most frequently altered oncogenes in brain cancers. Except for hematopoietic cells, the majority of cell types express ErbB family members [35].

In glioblastoma cells, the EGFR tyrosine kinase activity may be dysregulated by multiple oncogenic mechanisms, such as gene mutation, overexpression of EGFR protein, increased gene copy number, rearrangements of chromosomes, and activation by autocrine function [59].

#### 3.2.1. Mutations of Cell Signaling Regulators

The EGFR gene is located on chromosome 7p11.2 and consists of 28 exons encoding a transmembrane protein receptor composed of 464 amino acids. Exons 5–7 and 13–16 encode the ligand binding domain, and exons 18–24 encode the tyrosine kinase domain. The region encoded by exons 25–28 is the site of autophosphorylation.

Although EGFR is one of the most important drug targets in cancer therapies, its mutations present an organ–site asymmetry, depending on the cancer’s organ of origin [60]. Although mutations occur in the kinase domain (KD) in other tumors, in gliomas, heterogeneous mutations and deletions are focused on the ligand-binding ectodomain (ECD). This tissue-specific feature leads to type-II tyrosine kinase inhibitors (TKIs) with high sensitivity for the inactive symmetric KD dimer (sKD), when administered in GBM mutations [61]. However, both intra- and extracellular GBM mutations result in ligand-independent oncogenic activation.

Almost 50% of the tumors characterized by EGFR amplification are positive for the mutant EGFRvIII and EGFR single nucleotide variants (SNVs). Due to this tumor-specific feature, novel therapeutic agents are currently under development to target the overexpressed EGFR or EGFRvIII proteins. An in-frame deletion of exons 2–7 characterizes the EGFRvIII, which results in overexpression of a truncated receptor that lacks some significant parts of the ECD. This prototypic oncoprotein is unable to bind ligands, and it is constitutively active. Several studies examined the effect of the EGFRvIII constitutive activity on the wtEGFR and ErbB2 protein levels. For example, one study evaluated the effect of Tyrphostin AG1478 on the protein levels and demonstrated that its administration increased protein levels of wtEGFR and erbB2 in vIIIA1 cells, due to the catalytic activity of EGFRvIII, while in its absence, the levels were reduced [62]. Furthermore, the unique peptide sequence of EGFRvIII generated by the fusion of exons 1 and 8 may serve as a tumor-specific target in immunotherapy [63], although subsequent phase III trial results are not as promising as initially anticipated [64].

A meta-analysis performed in 2017 by Felsberg et al. proved that EGFRvIII and EGFR SNVs do not represent prognostic keys in EGFR-amplified glioma patients. However, the amplification of EGFR is retained in recurrent glioma [63], although improved long-term survival by EGFRvIII therapy has been reported in glioblastoma patients [65].

Nevertheless, the research on EGFRvIII continues, producing inconclusive results. For example, Struve et al. just published in early 2020 the results of a study focused on the effect of EGFRvIII in regulating DNA mismatch repair. They tested if EGFRvIII influences temozolomide’s sensitivity and demonstrated that, under standard treatment with temozolomide, EGFRvIII expression leads to prolonged survival only in patients with tumors with O6-methylguanine-DNA methyltransferase (MGMT) methylated promoter. Their results showed that EGFRvIII sensitizes a type of GBM to the current standard of care treatment with temozolomide through the upregulation of DNA mismatch repair (MMR) [65]. However, patients with tumors that have both EGFRvIII and MGMT methylation are very uncommon, and the conclusion that EGFRvIII status was associated with increased survival had a *p* = 0.06. This level would not normally be considered significant, especially not in this sort of multivariate analysis [66].

#### 3.2.2. Overexpression and Gene Amplification

The EGFR gene is amplified in approximately 40% of glioblastomas. The primary and secondary GBM differ in genetic profiles and primary GBMs have a higher prevalence of EGFR gene amplification and overexpression than secondary GBMs [67]. In a study performed by Watanabe et al., EGFR gene amplification was associated with protein overexpression in most tumor cells, but 10% of GBM with overexpression of EGFR protein lacked EGFR gene amplification [68]. However, previous studies have stated that EGFR overexpression or activation does not necessarily cause a simple amplification of its downstream signals, but dose-dependent changes in oncogene-induced downstream signaling and biological responses have been reported [69].

#### 3.2.3. Rearrangements of Chromosomes

Breakpoint sequence analyses proved different types of chromosomal rearrangements and mechanisms of DNA repair. Analyses of single nucleotide polymorphisms suggested that different deletions may appear from amplified non-vIII EGFR precursor [70].

In a study performed in 2018 on glioma tumor samples by Tomoyuki et al., complex chromosomal rearrangements involving chromosome 7 were observed [70].

A study performed by Lopez-Gines et al. showed that trisomy/polysomy 7 and monosomy 10 were frequently associated with glioma. The combination of these anomalies is important in glioblastoma’s tumorigenesis. Moreover, the association seems to be independent of EGFR gene amplification [71].

#### 3.2.4. Activation by Autocrine Function

It is well known that wild-type EGFR ligands such as transforming growth factor-alpha (TGF-alpha) and heparin-binding EGF (HB-EGF) are often increased in glioblastoma leading to an autocrine loop resulting in the autonomy growth of glioma cells [72]. GBM expresses an EGFR mutant (EGFRvIII) that signals constitutively, does not bind ligand, and is considered to have more tumorigenicity than wild-type EGFR. In a U251-MG glioma cell line, the expression of EGFRvIII may result in specific up-regulation of some genes (TGF-α, EPHA2, HB-EGF, IL8, FOSL1, MAP4K4, DUSP6, and EMP1) influencing signaling pathways involved in oncogenesis. TGF-α and HB-EGF (EGFR ligands) induce the expression of EGFRvIII, suggesting that EGFRvIII has a role in creating an autocrine loop with wild-type EGFR. By inhibiting HB-EGF activity with neutralizing antibodies, EGFRvIII-induced cell proliferation may be reduced, suggesting that EGFRvIII-HB-EGF-wild-type EGFR autocrine loop has a major role in signal transduction in glioblastoma cells [73]. Furthermore, studies have demonstrated that the expression of the EGFR alone has a poor transformation effect on cells. Though, coexpression of TGF-α ligand leads to a significant increase in transformation and therapies based on neutralizing the ligands have demonstrated the decreased growth of cells that harbor such loops [74,75].

## 4. Applied Theory—Therapies Targeting EGFR

The distribution of EGFR in cancer cells is the basic pillar of many targeted strategies pursued to inhibit its signaling pathway [76,77].

EGFR activity may be controlled by binding to the tyrosine kinase domain or binding to the extracellular component. There are three generations of tyrosine kinase inhibitors approved for clinical use. The first mechanism targets signal transduction and is characteristic of the tyrosine kinase inhibitors (RTKIs, TKIs), which bind to the tyrosine kinase domain of EGFR and inhibit its activity. First-generation TKIs, inhibit the receptor by competitive binding with ATP. Subsequent generations of TKIs were created to overcome drug resistance. Second-generation TKIs irreversibly inhibit all four ERBB (originally named because of the homology with the erythroblastoma viral gene product, v-erbB) receptors, whereas the third-generation TKI are specifically designed to target the T790M resistance mutation [78]. As first-generation inhibitors, active drugs include: erlotinib, gefitinib, lapatinib and vandetanib. Afatinib, dacomitinib, and tesevatinib are examples of second-generation small molecule EGFR inhibitors. Osimertinib is the first third generation RTKI. The monoclonal antibodies act as receptor blockers by binding to the extracellular component of the EGFR and block it from binding to its ligands. Cetuximab, necitumumab, and panitumumab are examples of biological therapy targeting EGFR [21]. An overview of clinical trials focused on anti-EGFR strategies used in GBM is provided in Table 1.

Other strategies consist of radio-immunotherapy, docking molecule conjugate toxins, chimeric antigen receptor T cells (CAR-T cells), RNA-based therapies, oncolytic viruses, exosomes, and nanoparticles [100]. EGFR-targeted nanoparticles may be combined with focused ultrasound to achieve local drug delivery [101]. Studies have shown that magnetic nanoparticles’ superparamagnetic properties allow them to be guided by an external magnet. However, their therapeutic use is limited in treating in vivo brain pathologies due to insufficient local ability to cross the blood–brain barrier. So, focused ultrasound combined with magnetic targeting synergistically delivers drug-loaded magnetic nanoparticles at the target tissue [102]. Boronated EGFR binding compounds are under investigation in so-called boron neutron capture therapy (BNCT). To improve the unsatisfactory bioavailability of large molecules or viruses due to low blood–brain barrier permeability, the convection technique (CED) is also being investigated [103]. Studies have shown that magnetic nanoparticles’ superparamagnetic properties allow them to be guided by an external magnet. However, their therapeutic use is limited in treating in vivo brain pathologies due to insufficient local ability to cross the blood–brain barrier. So, focused ultrasound combined with magnetic targeting synergistically delivers drug-loaded magnetic nanoparticles at the target tissue [101,102].

**Figure 2:**
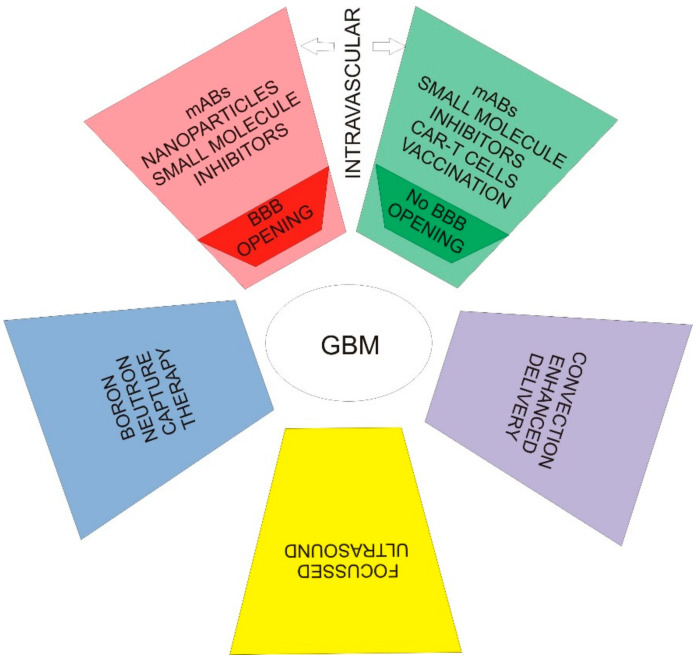
EGFR-based therapies in glioblastoma. (BBB—blood brain barrier, mABs—monoclonal antibodies, CAR-T—Chimeric anti-gen receptor T cell therapy)

For example, a study using Cetuximab conjugated magnetic iron oxide nanoparticles showed a significantly enhanced anti-tumor activity compared tocetuximab alone. This was due to improved cellular targeting and uptake, EGFR internalization, EGFR signaling alterations, and apoptosis induction in glioma stem-like cells and tumor non-stem cells that expressed EGFR [104]. In Figure 2, the EGFR-based therapies used in glioblastoma are mentioned.

### 4.1. Small Molecule Receptor Tyrosine Kinase Inhibitors

Although several compounds are approved for various diseases, none are approved for glioblastoma due to numerous negative clinical trials. Trials have not shown efficacy either alone or in combination for the oldest small molecule kinase inhibitors: gefitinib, erlotinib, lapatinib, and afatinib [105].

Gefitinib (Iressa, ZD1839) is the first approved EGFR-targeted small-molecule. Initial results from the clinical studies proved that gefitinib was safe when administered for lung carcinoma. However, responses were observed only in a subset of patients featuring chemotherapy–refractory advanced NSCLC (Nonsmall-cell lung carcinoma). The specific mutations of the EGFR gene explained this. It was suggested that these mutations stabilize the interaction of ATP and gefitinib with EGFR. Nevertheless, in the Phase II trial for recurrent GBM, gefitinib did not show improved overall survival [106], neither in the Phase I/II trial when combined with radiation in newly diagnosed GBM [107].

Erlotinib (Tarceva, OSI-774) proved to prolong NSCLC patients’ survival rate upon chemotherapy [108]. These results cannot be achieved in GBM, because EGFR mutations occur in the extracellular domain in GBM, whereas in lung cancers, they are typically observed in the kinase domain. Therefore, unlike NSCLC, GBMs are not sensitive to first-generation EGFR inhibitors [103]. As a single agent, it showed no efficacy in newly diagnosed GBM [109] and later studies that co-administered temsirolimus or bevacizumab were also unsuccessful [110,111].

Lapatinib (Tykerb, GSK 572016) had minimal efficacy alone or in combination with pazopanib in recurrent glioblastoma [112].

Afatinib (Tovok, BIBW2992) had limited efficacy as a single agent in one clinical trial in recurrent glioblastoma [79].

One of the drawbacks of the small molecule inhibitors is their brain penetrance. A study performed by Liu et al. showed that erlotinib could be distributed inside an intracranial U87 xenograft [113]. In another clinical trial, Gefitinib tissue concentration was two- to three-fold plasma concentrations, which was not the cause of insufficient efficacy [114].

Tesevatinib is another second-generation RTKI that is currently under evaluation in patients with recurrent GBM [92]. The first results should be published this year. This trial investigates the drug activity in EGFRvIII positive and negative GBM, with or without EGFR amplification.

Dacomitinib (Vizimpro, PF299804) is a second-generation EGFR inhibitor. Despite its poor global results in a phase II trial in recurrent GBM, Dacomitinib significantly benefited some patients [115].

A recent study investigating Osimertinib (AZD9291), a third-generation EGFR inhibitor, showed that it inhibits with high potency (<100 nM) the constitutive activity of EGFRvIII tyrosine kinase while also inhibiting its downstream signaling. Furthermore, Chagoya et al. proved that osimertinib inhibited the in vitro growth of the D317 cell line and heterotopic and orthotopic xenograft models [89].

To date, there have been eight completed clinical studies involving glioma and Vandetanib (Caprelsa, ZD6474), a second-generation EGFR inhibitor. They all investigated vandetanib’s effect together with other therapies (radiotherapy or therapeutic agents), but the results were not satisfactory [116].

### 4.2. Monoclonal Antibodies

Cetuximab (Erbitux, DTXSID70142901) is a monoclonal antibody (mAb) targeting the L2 domain of EGFR, preventing dimerization and subsequent cross-activation, thus interrupting downstream signal transduction. It has been approved for the treatment of colorectal, head, and neck cancers [117]. In the progressive high-grade glioma (HGG) patient population, the drug was well tolerated but had limited activity and failed to demonstrate benefit [118]. In new research, the photo-immunoconjugate nanoparticle (PIC-NP) significantly enhanced the photosensitizers in cancer cells and increased the light-activated cytotoxicity in U87 cells overexpressing EGFR [119,120].

Nimotuzumab (OSAG101) is another antibody targeting the L2 domain of EGFR. It was tested in clinical trials for its efficiency in adults with glioblastoma, but the results were not satisfactory. Currently, there is an ongoing clinical trial investigating the effect of nimotuzumab co-administered with temozolomide and radiotherapy. Preliminary results show that nimotuzumab was well tolerated, with an increased survival rate in newly diagnosed GBM patients [86].

Another antibody targeting the L2 domain of EGFR is Panitumumab (Vectibix, ABX-EGF). In combination with irinotecan, it was not very effective in solid tumors [121]. Panitumumab -IRDye800 is currently under investigation in Phase II trials as a GBM diagnostic agent [122].

bscEGFRvIIIxCD3 is a bispecific T-cell engager antibody (BiTEs) that binds to the CD3 T-cell coreceptor and recruits cytotoxic T cells. It was designed to redirect the T-cells towards tumors expressing EGFRvIII. Used in vitro and in vivo on mice, bscEGFRvIIIxCD3 showed the potent killing of GBM expressing EGFRvIII [123].

mAB806 is an antibody targeting the EGFRvIII-specific sequence. The antibody mAb806 is under investigation for glioblastoma treatment, although its mechanism of action remains unknown [124]. It was shown to potentiate the sensitivity of glioma xenotransplants to radiotherapy [125].

### 4.3. Targeted Isotopes

The isotope 125I mAB425 is a radioactive isotope conjugated with a specific antibody. Emrich et al. demonstrated that the administration of 125IMAb425 and intensive medical management led to a significant increase in median survival in patients with high-grade gliomas [126]. However, the results of subsequent studies failed expectations.

### 4.4. Immunotherapy

#### 4.4.1. CAR-T Cells Targeting EGFRvIII

A new technology developed in cancer therapy is the engineering of T cells to recognize their target by expressing a chimeric antigen receptor (CAR). Glioblastomas express the EGFRvIII, with its unique site of antigenicity. Therefore, these chimeric antigen receptor (CAR)-T cells were engineered to recognize the vIII-receptor mutation through a humanized single-chain antibody fragment (scFv) fused with some key constituents of T-cell receptor intra-cytoplasmic signaling domains [127].

This strategy is currently in early clinical trials. Sahin et al. developed a third-generation chimeric antigen receptor (CAR), specific for EGFRvIII (G3-EGFRvIII), that expresses CD28 (Cluster of Differentiation 28) and CD134 (Cluster of Differentiation 134). Their findings suggest that G3-EGFRvIII CAR represents a potential anti-glioblastoma strategy [128]. A Phase 1 pilot study that investigated the safety and feasibility of CAR-T-EGFRvIII in treating patients with EGFRvIII+ glioblastoma just terminated in 2019, and results are expected to be published [129]—currently, 15 trials are still recruiting.

#### 4.4.2. EGFR as an Immunologic Target—Vaccination

EGFRvIII represents the most common mutation of EGFR. It creates a tumor-specific antigen detectable in almost 30% of human GBM. Deleting the EGFR exons 2–7 results in EGFRvIII with a truncated extracellular domain, resulting in a unique, GBM cell-specific, antibody-reactive antigen. An EGFRvIII-specific peptide conjugated to a keyhole limpet hemocyanin represents the structure of a vaccine called Rindopepimut (CDX110). The latest Phase III clinical trial showed that rindopepimut did not increase the survival rate in newly diagnosed glioblastoma patients [130,131,132].

### 4.5. Targeting the Regulation of EGFR Gene Expression

This strategy consists of using antisense oligonucleotides, siRNA, ribozymes, and miRNA. In glioblastoma, the microRNAs control the post-transcriptional gene expression of receptor tyrosine kinase (RTK) signaling pathways by blocking or accelerating the mRNA. Recent work demonstrates that extracellular vesicles (EVs) can carry and transfer EGFR [133] and that cell communication through EVs enhances glioblastoma’s intratumoral heterogeneity [134]. Bronisz et al. showed that miR-1 could interact with a major EV protein Annexin A2 (ANAXA2) to reduce glioblastoma tumorigenicity [135]. Furthermore, one recent study of Liao et al. showed that the extracellular EC domain methylation using protein arginine methyltransferase 1 (PRMT1) increased EGF binding and dimerization, with enhanced receptor activation counteracting the effect of cetuximab in a mouse model of colon cancer [136]. The expression of the EGFRvIII is also under investigation. Unfortunately, none of the strategies targeting EGFR gene expression regulation have yet had any preclinical development.

### 4.6. Nanoparticles

In order to efficiently deliver the therapeutic agent, novel pharmaceutical formulations are currently used. It is well known that the bioavailability of drugs may be low because of the blood-brain barrier (BBB) permeability. Nanoparticles are vesicular carriers able to increase the bioavailability due to targeted drug release while protecting their content. EGFR is an ideal molecule for glioblastoma tumor targeting and numerous agents have been entrapped in a variety of nanoparticles [137].

There is only limited experience in early clinical trials for cetuximab conjugated liposomes [138]. A Phase II study just terminated in March 2020 investigated the effect of combining Temozolomide and a nanocomplex called SGT-53 (systemic gene therapy—53) to treat recurrent glioblastoma [139]. Previous results showed prolonged survival rates in glioblastoma mouse models [140].

Regardless of strategy, all therapeutic agents face the main problem of delivery across the blood–brain barrier, often cited as the explanation for EGFR targeting failure in glioblastoma [103].

## 5. Facing a Real Challenge—Drug Resistance

There is evidence that targeted therapy towards mutations responsible for cancer growth and progression is effective in different tumor types. For GBM, the responses to EGFR-pathway inhibitors were not as expected, and they are mainly explained by drug resistance [141]. Two major mechanisms could explain the EGFR therapy resistance.

The first resistance mechanism involves target independence. In this situation, glioma cells without EGFR protein expression experience no negative impact from EGFR inhibitors. For this type of resistance, the loss of extra-chromosomally encoded EGFR is a frequent mechanism. Target independence may occur after small molecule therapy. The dynamic EGFRvIII expression regulation by small circular fragments of extra-chromosomal DNA is involved in the resistance to EGFR inhibition. Some studies demonstrated that GBM cells treated with erlotinib reversibly suppressed mutant EGFR by producing extra-chromosomal DNA, making the GBM cells resistant to EGFR inhibition. After withdrawing erlotinib, the mutations re-emerged on extra-chromosomal DNA, leading to the upregulation of EGFRvIII with consequent re-sensitization of GBM cells [142].

The second mechanism regards target compensation. In this situation, glioma cells fight back against EGFR inhibition by activating compensatory pathways independent of EGFR signaling. Insulin-like growth factor 1 (IGFR1), platelet-derived growth factor β (PDGFβ), mesenchymal-epidermal transition (cMET), and their downstream targets are involved in these compensatory pathways [77].

Given these two mechanisms, rational strategies should include multi-target therapies targeting truncation mutations for the first mechanism and multi-target therapies targeting compensatory proteins for the second mechanism. Resistance may be overcome by dosing/epigenetic therapy, targeting truncation mutations, or through multitarget therapy.

For the first strategy, glioma cells’ re-sensitization may be achieved by using drug scheduling [143]. Pulsatile intermittent drug therapy with EGFR-inhibitors in high doses can lead to better inhibition of the target, delay of therapy resistance, and toxicity reduction compared to continuous dosing [144].

Targeting truncated mutations is a suitable strategy for glioma treatment due to their high frequency in this pathology. As candidates for co-targeting, the following deserve discussion: PTEN with PI3K as a molecular target and cyclin-dependent kinase inhibitor 2 A (CDKN2A) with cyclin-dependent kinase (CDK)4/6 as a molecular target. Some PI3K inhibitors are currently undergoing clinical trials, such as GDC-0084, PX-866, pilaralisib, buparlisib, and XL765 [145]. Abemaciclib, palbociclib, and ribocyclib are examples of FDA (Food and Drug Administration)-approved oral drugs with good BBB permeability [146,147] that may be investigated for targeting CDK4/6.

In many types of cancer, multitarget therapy is a preferred option. Therapies may become more efficient by combining EGFR inhibitors with other downstream blocking agents. Several glioma specific epitopes such as IL13RA2 and EphA2 are under investigation for poly-target therapy with antibody drug conjugates (ADCs) and CAR-T cells [148,149,150,151].

In a recent 2020 study, Meng et al. proved that the cross-activation of EGFR and MET signaling pathways contributes to temozolomide resistance in glioblastoma patients. To simultaneously diminish both EGFR and MET activation, they developed a nanoinhibitor with double functionalized brain-target (BIP-MPC-NP) by conjugating cMBP and Inherbin3 modified poli-2-methacryloyloxyethyl phosphorylcholine (MPC)-nanoparticles. The study shows that DNA damage repair is reduced, and sensitivity is augmented by downregulating the E2F1 transcription factor in temozolomide resistant glioma in mice. These results demonstrate that the abovementioned nanoinhibitor is a suitable candidate for overcoming drug resistance in glioma [152].

Furthermore, patient mutations affecting the trafficking of therapeutic antibodies is another potential mechanism contributing to therapeutic resistance [153,154].

## 6. Conclusions

The global understanding of EGFR signaling has dramatically advanced in the last ten years. However, extensive work is still required in order to understand all signaling pathways and their implications fully. The application of EGFR-targeted therapy for glioma treatment has been less successful than expected. Gliomas require a complex signaling network that dictates the tumor sensitivity of EGFR-targeted therapies. Low BBB penetration, as well as tumor heterogeneity, secondary mutations, and compensatory signaling pathways, contribute to resistance. The development of new combinatorial therapies may improve patient quality of life through personalized, tailored choices of appropriate therapeutic strategies. An integrated approach is required to offer a complete view of this critical receptor by combining cellular, biochemical, structural, and genetic modeling techniques.

## Figures and Tables

**Table 1:** Epidermal growth factor receptor (EGFR)-targeted therapies for adult high-grade gliomas currently in investigational and/or clinical use.

Therapeutic Agent	Mechanism	Results	Reference
Afatinib (Tovok, BIBW2992)	Second-generation EGFR inhibitor	As a single agent, Afatinib proved good safety, but limited activity on GBM patients. It was promising in combination with TMZ in a case report.6 months progression-free survival (PFS) worse than TMZ: Afatinib alone 3% vs. Afatinib + TMZ 10% vs. TMZ alone 23% Ongoing clinical trials: NCT02423525	[79]
Cetuximab (Erbitux, DTXSID70142901)	Antibody targeting the L2 domain of EGFR	Cetuximab was not very effective in GBM clinical trials. 6-month PFS was 33%, and median PFS was 16 weeks Ongoing clinical trials: NCT02800486 NCT02861898	[80]
Dacomitinib (Vizimpro, PF299804)	Second-generation EGFR inhibitor	Dacomitinib proved to be promising in pre-clinical models. 6-months PFS was 10.6% with a median PFS of 2.7 months Ongoing clinical trials: NCT01112527 NCT01520870	[81]
Erlotinib (Tarceva, OSI-774)	First-generation EGFR inhibitor	Erlotinib showed poor results in GBM clinical trials. The median PFS: 1.8 months Erlotinib vs. 2.4 months TMZ/BCNU (bis-chloroethylnitrosourea) Ongoing clinical trials: NCT01257594 NCT02239952	[82]
Gefitinib (Iressa, ZD1839)	First-generation EGFR inhibitor	Gefitinib showed poor results in GBM clinical trials. The median overall survival time from treatment initiation was 39.4 weeks	[83]
Lapatinib (Tykerb, GSK 572016)	First-generation EGFR inhibitor	Lapatinib demonstrated poor results in GBM clinical trials. The studies lacked objective responses, with early progression rate of 76%. Ongoing clinical trials: NCT01591577 NCT02101905	[84]
Nimotuzumab (OSAG101)	Antibody targeting the L2 domain of EGFR	Nimotuzumab in addition to standard treatment is well tolerated and has increased survival rates in EGFR positive expression newly diagnosed GBM patients. The PFS and OS rates were 49.3% and 83.3% for 1-year and 29.0% and 51.1% for 2-year. Ongoing clinical trials: NCT03620032	[85]
Osimertinib (AZD9291)	Third-generation EGFR inhibitor	Osimertinib is in phase I/II clinical trial. Compared to other EGFR-TKIs, AZD9291 demonstrated improved ability to inhibit GBM cells proliferation.Complete response of left frontal lobe tumor after 4 weeks of osimertinib.	[86,87,88]
Panitumumab (Vectibix, ABX-EGF)	Antibody targeting the L2 domain of EGFR	Panitumumab was not very effective in GBM clinical trials.Panitumumab-IRDye800CW specificities for tumor core and margin were slightly higher than those of 5-ALA. Ongoing clinical trials: NCT03510208	[89]
Rindopepimut (CDX110)	Vaccine	When co-administrated with Bevacizumab, Rindopepimut significantly prolonged patient survival. 6 months PFS was 28% (rindopepimut), compared with 16% (control)Phase II trial (NCT00458601) was completed in 2018.	[90]
Vandetanib (Caprelsa, ZD6474)	Second-generation EGFR inhibitor	Vandetanib was a moderately tolerated drug, with no significant activity as a single agent in patients with recurrent malignant glioma. Median overall survival was 6.3 months. Ongoing clinical trials: NCT02239952	[91]
Tesevatinib (KD019)	Second-generation EGFR inhibitor	Tesevatinib is in Phase II study in patients with recurrent glioblastoma, with no results posted. Ongoing clinical trials: NCT02844439	[92]
bscEGFRvIIIxCD3	Antibody BisAbs	Fully human bispecific single chain antibody fragments bi-scFv (EGFRvIII:CD3 bi-scFv) was recently developed with the aim to redirect CD3-expressing T cells to target malignant EGFRvIII-expressing glioma.	[93]
mAB806	Antibody targeting the EGFRvIII-specific sequence	Structural extracellular mutations lead to a similar intermediate conformation, that can be synergistically targeted intra- and extracellularly by mAb806 antibody. Lapatinib co-treatment sensitized unresponsive wild type (WT)-EGFR to mAb806.	[94]
^125^I mAB425	Antibody toxin or radioactive isotope conjugated	Single or in combination with TMZ, ^125^I mAB425 prolonged patient survival (median survival of 20.4 months, compared to 14.5 months for ^125^I mAB425 alone), with minimal toxicity in normal tissue.	[95]
Chimeric anti-gen receptor T cell therapy (CAR-T cells)	Chimeric antigen receptor therapy (CARs) targeting EGFRvIII	Chimeric antigen receptor (CAR) T cells are in phase I clinical trials in high-grade glioma (HGG) patients. Pre-clinical models proved to be promising. Ongoing clinical trials: NCT02331693 NCT02844062 NCT02209376 NCT01454596 NCT02664363	[96,97]
Antisense oligonucleotides, siRNA, ribozymes, and miRNA-based therapy	RNA-based therapies	Feasibility of RNA-based therapies must be further evaluated using pre-clinical models.	[98,99]

## Data Availability

The data presented in this study are openly available.
